# Prior-FOVNet: A Multimodal Deep Learning Framework for Megavoltage Computed Tomography Truncation Artifact Correction and Field-of-View Extension

**DOI:** 10.3390/s25010039

**Published:** 2024-12-25

**Authors:** Long Tang, Mengxun Zheng, Peiwen Liang, Zifeng Li, Yongqi Zhu, Hua Zhang

**Affiliations:** 1 School of Biomedical Engineering, Southern Medical University, Guangzhou 510515, China; tlong012@163.com (L.T.); ngc2237_rose@163.com (M.Z.); pw87672155@gmail.com (P.L.); crazy_li_15@163.com (Z.L.); 13066456876@163.com (Y.Z.); 2 Guangdong Provincial Key Laboratory of Medical Image Processing, Southern Medical University, Guangzhou 510515, China; 3 Guangdong Province Engineering Laboratory for Medical Imaging and Diagnostic Technology, Southern Medical University, Guangzhou 510515, China

**Keywords:** megavoltage computed tomography, truncation artifacts, field-of-view extension, contrastive learning, swin transformer

## Abstract

Megavoltage computed tomography (MVCT) plays a crucial role in patient positioning and dose reconstruction during tomotherapy. However, due to the limited scan field of view (sFOV), the entire cross-section of certain patients may not be fully covered, resulting in projection data truncation. Truncation artifacts in MVCT can compromise registration accuracy with the planned kilovoltage computed tomography (KVCT) and hinder subsequent MVCT-based adaptive planning. To address this issue, we propose a Prior-FOVNet to correct the truncation artifacts and extend the field of view (eFOV) by leveraging material and shape priors learned from the KVCT of the same patient. Specifically, to address the intensity discrepancies between different imaging modalities, we employ a contrastive learning-based GAN, named TransNet, to transform KVCT images into synthesized MVCT (sMVCT) images. The sMVCT images, along with pre-corrected MVCT images obtained via sinogram extrapolation, are then input into a Swin Transformer-based image inpainting network for artifact correction and FOV extension. Experimental results using both simulated and real patient data demonstrate that our method outperforms existing truncation correction techniques in reducing truncation artifacts and reconstructing anatomical structures beyond the sFOV. It achieves the lowest MAE of 23.8 ± 5.6 HU and the highest SSIM of 97.8 ± 0.6 across the test dataset, thereby enhancing the reliability and clinical applicability of MVCT in adaptive radiotherapy.

## 1. Introduction

The tomotherapy system, primarily focusing on intensity-modulated radiation therapy (IMRT), represents one of the most advanced modalities in contemporary oncologic radiotherapy [1,2]. This system uniquely integrates megavoltage fan-beam computed tomography (MVCT) technology, providing a powerful tool for precise tumor treatment [3]. MVCT images are typically obtained before treatment to ensure precise patient positioning, enabling accurate tumor targeting. Additionally, MVCT is employed in adaptive radiotherapy (ART) to revise treatment plans based on observed changes in tumor size, shape, or position [4].

**Figure 1:**
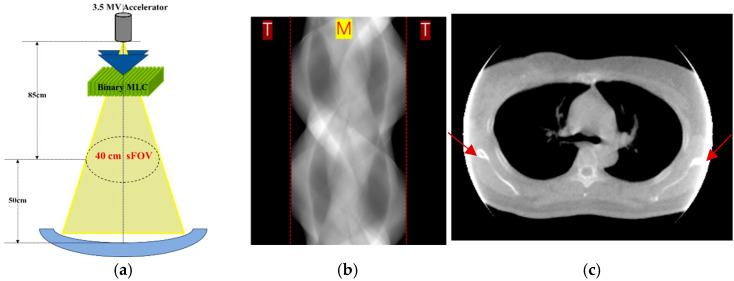
(**a**) The imaging geometry of MVCT. (**b**) An example of a truncated sinogram. The measured data are labeled “M”, and the truncated data, labeled “T”, are typically filled with zeros when uncorrected. (**c**) An MVCT image exhibiting truncation artifacts, where the blooming effect caused by truncation is indicated by the red arrows.

The scan field of view (sFoV) of modern CT systems typically ranges from 50 to 70 cm, ensuring that patients are fully covered during a CT scan. However, for MVCT imaging, the sFoV is limited to only 40 cm. This reduced coverage means that patients with a higher body mass index or external reference markers may not be entirely included within the sFoV during an MVCT scan, leading to projection data truncation. This truncation often results in abrupt changes in signal intensity at the sFOV boundaries. As shown in Figure 1, when using the filtered back-projection (FBP) reconstruction algorithm, these abrupt intensity changes are transferred and amplified in the reconstructed CT images, causing a blooming effect near the intersection of the patient’s body and the outer boundary of the FOV. Truncation artifacts in MVCT can significantly affect the accuracy of image registration with the planned kilovoltage computed tomography (KVCT), due to distortions and inconsistencies in anatomical structures. Additionally, the effectiveness of MVCT-based adaptive planning is hindered by the incomplete anatomical information in MVCT images. Consequently, addressing the challenge of truncation artifacts is vital for enhancing the reliability and clinical applicability of MVCT images.

In recent years, numerous research methods have been proposed for CT truncation artifact correction [5,6]. These approaches, including heuristic extrapolation methods such as symmetric mirroring [7], cosine function extrapolation [8], and water cylinder fitting [9], have been widely employed to correct projection data truncation. Li et al. observed that the water cylinder fitting method often introduces high noise fluctuations [10], leading to anatomical boundary artifacts, particularly in spiral scanning. To mitigate this issue, they proposed a roughness penalty function based on the Huber regularization function, which enhances the consistency of longitudinal boundaries. Xu et al. presented a method that applies the Helgason–Ludwig (HL) consistency condition in combination with a maximum likelihood statistical approach to restore truncated sinogram data before conducting FBP reconstruction [11]. These extrapolation techniques aim to achieve a smooth transition between the measured and truncated regions, alleviating the cupping artifacts within the FOV. To further address the truncation artifacts caused by the global filtering step in the FBP algorithm, several studies have proposed analytic reconstruction algorithms with alternating local filtering [12]. This approach decomposes the ramp filter into a local Laplacian filter and a non-local low-pass filter. Additionally, an interior tomography method known as differentiated back-projection (DBP) has been proposed [13,14], which utilizes prior knowledge to achieve theoretically accurate solutions. With the advent of compressed sensing techniques, iterative reconstruction methods based on total variation (TV) regularization have become the mainstream approach for CT reconstruction in cases of insufficient data [15,16]. However, these methods present significant limitations regarding model complexity, parameter variability, and residual image artifacts. They improve image quality within the FOV but fail to accurately reconstruct anatomical structures outside the FOV.

Meanwhile, significant progress has been made in deep learning methods for truncation artifact reduction. For instance, Huang et al. introduced an extended-field-of-view reconstruction method that integrates U-Net with an iterative reconstruction algorithm based on TV minimization [17]. However, this approach still faces challenges in robustness, especially in scenarios with very limited data and noise, and its dependence on the iterative TV framework increases overall complexity. Fournié et al. proposed a method that first performs sinogram extrapolation along the channel direction [18], followed by the use of a U-Net to reduce artifacts. Fonseca et al. introduced the HDeepFoV algorithm [19], which combines deep learning-based eFoV reconstruction with the HDFoV algorithm. The generated data are merged with the measured data within the sFOV of the CT scanner for final image reconstruction. Khural et al. compared generic CNNs [20], autoencoders, and U-Net architectures for generating the extended-field-of-view images, finding that the U-Net architecture achieved the lowest mean absolute error compared to the ground truth, thereby providing the most favorable results. Gao et al. proposed a transformer-based dual-domain network that combines recovery in both the image and sinogram domains to reconstruct complete CBCT images from truncated sinograms [21]. Huang et al. proposed a plug-and-play (PnP) method that integrates data consistency for measured data and learned prior image information for truncated data [22].

The aforementioned deep learning approaches have introduced several compelling models for truncation correction. However, these models often concentrate solely on the target image itself and lack prior knowledge of the material and shape of the truncated portion of the object. Therefore, the quality and fidelity of the obtained results are limited. Utilizing reference images as auxiliary information to achieve improved results is a well-established practice in numerous computer vision tasks, such as image super-resolution [23,24], deblurring [25,26], and denoising [27,28]. In clinical practice, KVCT is first obtained to design the treatment plan for tomotherapy, while MVCT is scanned just before treatment. Given that MVCT and planned KVCT images originate from the same patient and share identical anatomical structures, our research aims to utilize KVCT as prior information to enhance the recovery of MVCT images. In this study, we introduce Prior-FOVNet, a method designed to correct truncation artifacts and extend the field of view using the prior information derived from KVCT images. However, effectively leveraging the material and structural information from KVCT requires addressing intensity discrepancies between KVCT and MVCT images, which arise due to differences in imaging modalities. To bridge this gap, we employ a contrastive learning-based GAN to transform KVCT images into synthesized MVCT images, which serve as prior information for correcting MVCT in the image domain. The truncated MVCT, after undergoing sinogram domain extrapolation, is integrated with the synthesized MVCT images within a Swin Transformer-based image inpainting network, ultimately producing the corrected MVCT images. The main contributions of this work can be summarized as follows:

(1) Introduced the Prior-FOVNet framework, which leverages prior information from KVCT images to correct truncation artifacts and extend the field of view in MVCT.
(2) Utilized a contrastive learning-based generative adversarial network to transform KVCT images into synthesized MVCT images, addressing intensity discrepancies between modalities while preserving anatomical details.
(3) Employed a Swin Transformer-based inpainting network to reconstruct anatomical structures outside the truncated field of view, efficiently integrating local and global features for improved structural consistency.
(4) Experimental results demonstrate that Prior-FOVNet outperforms traditional and deep learning methods in artifact reduction and structural detail reconstruction, showing robust generalization across various truncation levels.

## 2. Method

### 2.1. Initial Truncation Artifact Correction

A basic sinogram domain extrapolation is first applied to mitigate the blooming effect (bright shading) in the pixels near the intersection between the patient’s body and the outer boundary of the sFOV. In this study, the algorithm proposed in reference [9] was used, which used the magnitudes and slopes of the projection samples at the location of truncation to estimate water cylinders that can best fit the projection data outside the SFOV.

**Figure 2:**
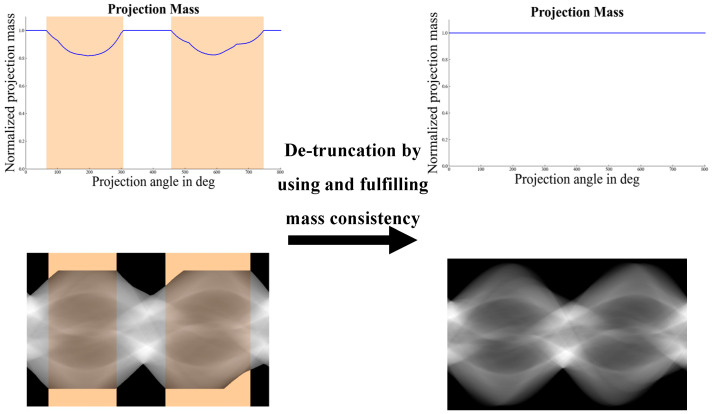
Sinogram extrapolation to ensure mass consistency across the sinograms. The left side displays the normalized sinogram with truncation, while the right side illustrates that consistency is achieved following the extrapolation. The orange areas denote the truncated regions where the normalized mass falls below 1.

As illustrated in Figure 2, the core principle of this algorithm is based on the consistency of mass in the sinogram along the detector direction. The key aspect of this method lies in accurately determining the position and radius of the cylindrical region:

(1) $$x\left(k\right)=\frac{s\left(k\right)p\left(k\right)}{4\mu_{w}^{2}},and\;R\left(k\right)=\sqrt{\frac{p^{2}(k)}{4\mu_{w}^{2}}+x^{2}(k)},$$

where p(k) and s(k) refer to the projection amplitude and slope at the truncated edges, respectively. The parameters x(k) and R(k) represent the position and radius of the fitted cylindrical structure. The attenuation coefficient of water is denoted as $\mu_{w}$. The use of a water-equivalent cylindrical model is computationally efficient. Water cylinder extrapolation (WCE) facilitates the smooth extension of missing regions and effectively reduces artifacts along the boundaries.

### 2.2. Synthesis of MVCT Image with TransNet

**Figure 3:**
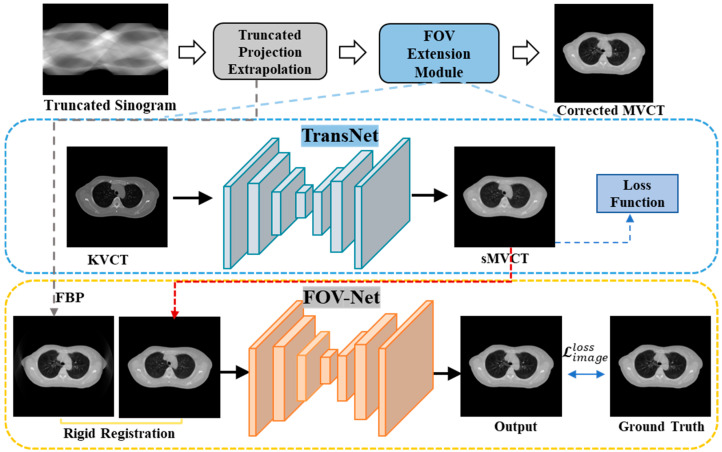
The overall flowchart of Prior-FOVNet. First, an initial truncation correction is performed using the mass consistency condition and the water cylinder approach to extrapolate the missing data in truncated projections. Simultaneously, the KVCT images are processed by TransNet, which is a contrastive learning-based GAN to generate synthesized MVCT (sMVCT) images with matched intensity values with real MVCT. Finally, the pre-corrected MVCT and synthesized MVCT images are rigidly registered and fed into FOV-Net, which is a Swin Transformer-based image inpainting network that can integrate them to reconstruct the eFOV images.

**Figure 4:**
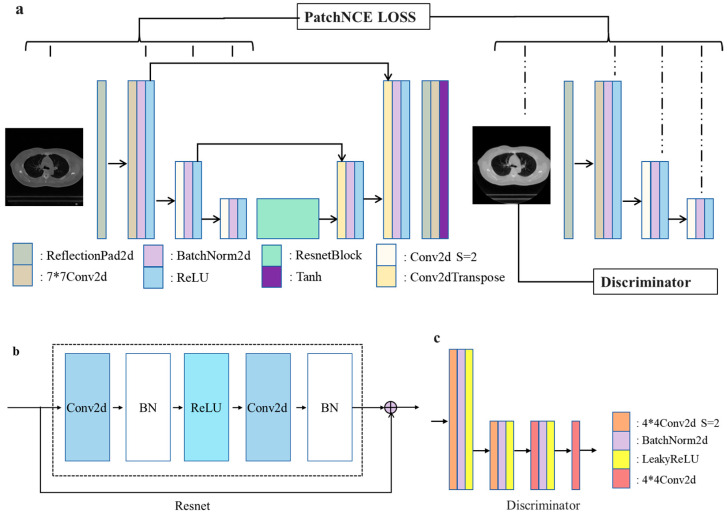
The architecture of TransNet is structured as follows: (**a**) illustrates the design of the encoder and decoder within the generator. (**b**) represents the ResNet block, a critical element that bolsters feature learning by leveraging residual connections. (**c**) elaborates on the architecture of the discriminator, responsible for distinguishing generated data from real data.

In the image-to-image translation task illustrated in Figure 3, our objective is to generate sMVCT images that closely resemble real MVCT images in terms of intensity and appearance while accurately preserving the structural content of the input KVCT images. In this work, we employed a GAN architecture based on contrastive learning named TransNet to implement sMVCT synthesis [29]. The network architecture is shown in Figure 4.

The generator function G is structured with two components: the encoder $G_{enc}$ and the decoder $G_{dec}$. The encoder processes the input image and produces a latent vector through a series of downsampling convolutional layers and residual bottleneck layers. The decoder, which takes this latent vector as input, reconstructs the output image. It consists of residual bottleneck layers followed by upsampling layers.

The model includes one discriminator $D_{MV}$, which is trained to learn the distribution of real MVCT images to distinguish from processed KVCT (sMVCT) images. The generator and discriminator are intertwined through adversarial loss [30,31] to encourage the output to visually resemble images from the target domain, as follows:

(2) $$L_{GAN}\left(G,D,X,Y\right)=E_{y\sim Y}\log D\left(y\right)+E_{x\sim X}\log\left(1-D\left(G\left(X\right)\right)\right),$$

**Figure 5:**
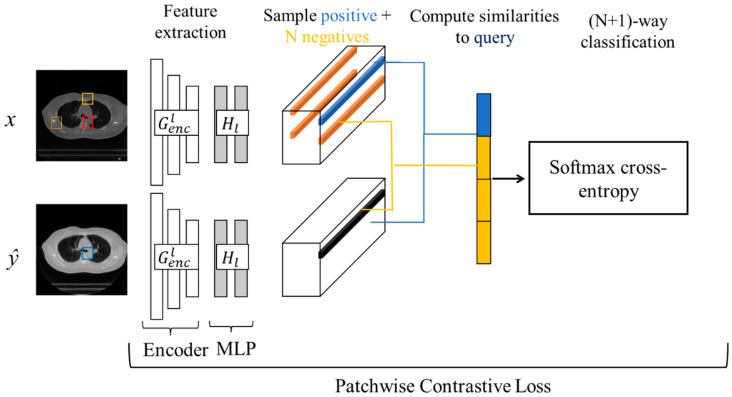
The flowchart of patchwise contrastive loss design.

To prevent distortions in anatomical structures and ensure consistency between the original and translated images, it is crucial to introduce an additional loss term that serves as an alternative to the cycle consistency loss. In this study, we focus on preserving structural consistency between domains by integrating contrastive learning into the GAN framework [32], using the patchwise contrastive loss. Compared to cycle consistency loss, this approach imposes lower requirements on the dataset, as it does not necessitate a bijective mapping between the two domains. Additionally, it more accurately preserves the critical features and structures of the input images, resulting in superior image quality. As illustrated in Figure 5, after processing the images through the encoder $G_{enc}$, we extract a feature stack composed of multiple layers. The PatchNCE loss used within the contrastive learning framework involves selecting certain layers of interest from these feature stacks. The feature maps from the selected layers are then passed through a two-layer MLP network, after which patches are randomly selected from these layers. As depicted in Figure 5, we sample a query patch from the output and compare it to the input patch at the same location. We set up an (N + 1)-way classification problem, where N negative patches are sampled from the same input image at different locations. The query, positive, and N negatives are, respectively, mapped to k-dimensional vectors $v,\;v^{+}\in R^{K}$, and $\;v^{-}\in R^{N\times K}$. Here, $v_{n}^{-}\in R^{K}$ represents the n-th negative. The vectors are normalized to the unit sphere to prevent spatial collapse or expansion. An (N + 1)-way classification problem is established, where the distances between the query and other examples are scaled and passed as logits. The loss function is then computed in the form of cross-entropy loss.

(3) $$l\left(v,v^{+},v^{-}\right)=-\log\left[\frac{\exp\left(\frac{v\cdot v^{+}}{\tau }\right)}{\exp\left(\frac{v\cdot v^{+}}{\tau }\right)+\sum_{n=1}^{N}\exp\left(\frac{v\cdot v_{n}^{-}}{\tau }\right)}\right],$$

where $\exp\left(v\cdot v^{+}\right)$ denotes the cosine similarity between the feature representations of patches v and $v^{+}$, and τ is the temperature parameter governing the distribution’s sharpness. The loss function aims to maximize the similarity between positive pairs while minimizing the similarity between the chosen negative pairs.

The loss is computed for each of the layers and summed together. This constitutes the PatchNCE loss we employ.

(4) $$L_{PatchNCE}\left(G,H,X\right)=E_{x∼X}\sum_{l=1}^{L}\sum_{s=1}^{S_{l}}l\left({\hat{z}}_{l}^{s},z_{l}^{s},z_{l}^{S\backslash s}\right),$$

where ${\left\{z_{l}\right\}}_{L}={\left\{H_{l}(G_{enc}^{l}(x))\right\}}_{L}$ refers to the feature stack of the input image passed through the MLP layers, where $G_{enc}^{l}$ represents the output from the l-th selected layer. The layer index is denoted as l∈{1,2,…,L}, and $s\in \{1,\ldots ,S_{l}\}$, where s represents the spatial position, and $S_{l}$ indicates the number of spatial positions in each layer. The feature corresponding to the output image is denoted as ${\hat{z}}_{l}^{s}$. The feature of the input image is referred to as $z_{l}^{s}$, while $z_{l}^{S\backslash s}$ represents the remaining features.

The third loss is identity loss. The PatchNCE loss can be applied to images of domain y to prevent the generator from unnecessary changes. This loss essentially functions as a learnable, domain-specific identity loss.

Based on the above, the total loss of the TransNet is expressed as follows:

(5) $${L=L}_{GAN}\left(G,D,X,Y\right)+L_{PatchNCE}\left(G,H,X\right)+L_{PatchNCE}\left(G,H,Y\right),$$

### 2.3. Field-of-View Extension with FOV-Net

To further enhance image quality by extending the FOV and filling in the missing structural information outside the FOV, we propose integrating the synthesized sMVCT images into the corresponding FOV extension module. The pre-corrected image $I_{prec}$ generated through sinogram domain data extrapolation and the synthetic image $I_{sMV}$, generated by the TransNet, are input into the image inpainting network FOV-Net to produce the field-of-view extended image $I_{eFOV}$. The process is expressed as follows:

(6) $$I_{eFOV}=G_{FOV-Net}\left(I_{Prec},I_{sMV}\right),$$

The sMVCT is rigidly registered to the pre-corrected MVCT image before inputting the images into the FOV-Net.

**Figure 6:**
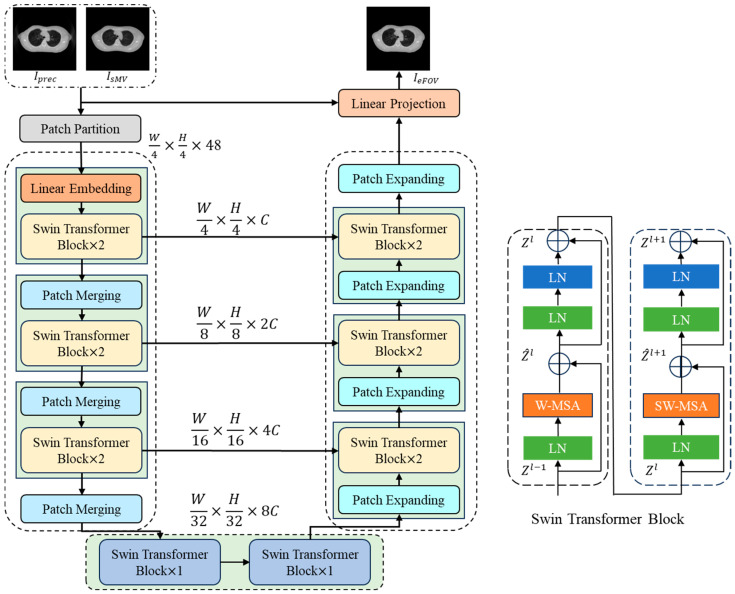
Architecture of the FOV-Net.

Figure 6 illustrates the architecture of the FOV-Net, which integrates the strengths of the Swin Transformer and the U-Net architecture [33]. In the encoder, the input is partitioned into non-overlapping 4 × 4 patches, which are then linearly mapped into 128 × 128 × 48 patch tokens. These tokens are processed through multiple Swin Transformer blocks and patch merging layers to generate hierarchical feature representations. The patch merging layer is responsible for downsampling and dimension increase, while the Swin Transformer block is responsible for feature representation learning. In the decoder, multi-scale features from the encoder are input into the Swin Transformer blocks of the decoder through skip connections to compensate for the spatial information loss caused by downsampling and to upsample the feature maps using patch expansion layers. Given the substantial overlap between the output and input images, skip connections are employed to copy the input image directly to the final linear layer.

The Swin Transformer block used in this work includes LayerNorm (LN), Window-based Multi-Head Self-Attention (W-MSA), residual connections, and a two-layer Multi-Layer Perceptron (MLP). As shown in the right of Figure 6, two consecutive Swin Transformer blocks are depicted. The W-MSA module and the Shifted Window-based Multi-Head Self-Attention (SW-MSA) module are applied to the two consecutive Transformer blocks, respectively. The window size is set to 8 × 8. The two consecutive Swin Transformer blocks can be represented as follows:

(7) $${\hat{z}}^{l}=W-MSA\left(LN\left(z^{l-1}\right)\right)+z^{l-1},$$

(8) $$z^{l}=MLP\left(LN\left({\hat{z}}^{l}\right)\right)+{\hat{z}}^{l},$$

(9) $${\hat{z}}^{l+1}=SW-MSA\left(LN\left(z^{l}\right)\right)+z^{l},$$

(10) $$z^{l+1}=MLP\left(LN\left({\hat{z}}^{l+1}\right)\right)+{\hat{z}}^{l+1},$$

where $z^{l}$ and $z^{l+1}$ denote the outputs of the W-MSA and MLP of the *l*-th block, respectively. The calculation method for self-attention is as follows:

(11) $$Attention\left(Q,K,V\right)=SoftMax\left(\frac{QK^{T}}{\sqrt{d}}+B\right)V,$$

where $Q,K,V\in R^{M^{2}\times d}$ represent the query, key, and value matrices, respectively. $M^{2}$ and d denote the number of patches in the window and the dimension of the query or key, respectively. The values in B are taken from the bias matrix $B\in R^{\left(2M+1\right)\times \left(2M-1\right)}$.

The loss function of the FOV-Net network acing as an image reconstruction loss L can be expressed as follows:

(12) $$L=||I_{eFOV}-I_{gt}||,$$

where ${\left\|\cdot \right\|}_{1}$ represents the L1 norm, and $I_{gt}$ denotes the ground truth image.

### 2.4. Implementation Details

In this work, all experiments were implemented using Python 3.6 based on the PyTorch 1.7.0 framework, running on an NVIDIA TITAN X GPU manufactured by NVIDIA Corporation, located in Santa Clara, CA, USA. The Adam optimizer was employed to optimize all network parameters [34]. The TransNet network was trained for 400 epochs with a batch size of 1. An initial learning rate of 2 × 10^−4^ was used for the first 200 epochs, which linearly decayed to zero over the following 200 epochs. The FOV-Net network was trained for 100 epochs with a mini-batch size of 8. An initial learning rate of 2 × 10^−4^ was applied for the first 80 epochs and then linearly decayed to zero over the subsequent 20 epochs. The Prior-FOVNet model contained about 15.7 million parameters.

## 3. Experimental Data and Evaluation Metrics

### 3.1. Experimental Data

In this study, the projection data of MVCT and the corresponding planning KVCT images of 38 patients were collected for network training. The data collection process strictly adhered to the principles of informed consent from patients. The data included images of the chest and pelvis, for a total of 912 slices. The original MVCT projection data were acquired using the Radixact^®^ treatment delivery system at a voltage of 3.5 MV, while the KVCT images were obtained using a SIEMENS CT scanner operating at 120 kV, manufactured by Siemens Corporation in Munich, Germany. For the TransNet training, the MVCT images reconstructed from the selected projection data without truncation were used. All MVCT images were reconstructed by using the filtered back-projection reconstruction algorithm with a size of 512 × 512. To simulate truncated MVCT images with different levels, projection data truncation with 30, 45, and 60 detector numbers was implemented. MVCT images were then reconstructed with the truncated projection data. For improved training efficacy of the TransNet and FOV-net, binary masks containing only the patient’s body structures were generated through thresholding, with the background and non-anatomical structures such as the examination table removed. Additionally, data augmentation was performed using affine transformations. In the testing phase, the projection data of MVCT for 10 patients, along with their corresponding planning KVCT images, were collected separately. These data contain some truncated projections, mainly in the chest and pelvis regions.

### 3.2. Evaluation Metrics

In addition to visually assessing the corrected MVCT, a quantitative evaluation of the image quality was conducted. The mean absolute error (MAE), root mean square error (RMSE), and structural similarity index (SSIM) were calculated between the generated images and the ground truth images within the test dataset.

(13) $$MSE(X_{eFOV},X_{gt})=\frac{1}{n}\sum_{i=0}^{n-1}{\left[X_{eFOV}(i)-X_{gt}(i)\right]}^{2},$$

(14) $$RMSE=\sqrt{MSE},$$

(15) $$MAE(X_{eFOV},X_{gt})=\frac{1}{n}\sum_{i=1}^{n}\left|X_{eFOV}(i)-X_{gt}(i)\right|,$$

(16) $$SSIM\left(X_{eFOV},X_{gt}\right)=\frac{\left(2\mu_{eFOV}\mu_{gt}+c_{1}\right)\left(2\sigma_{eFOV,gt}+c_{2}\right)}{\left(\mu_{eFOV}^{2}+\mu_{gt}^{2}+c_{1}\right)\left(\sigma_{eFOV}^{2}+\sigma_{gt}^{2}+c_{2}\right)},$$

where $X_{eFOV}(i)$ and $X_{gt}(i)$ represent the pixel values at the i-th position of the extended image and the corresponding ground truth image, respectively. The total number of pixels is denoted by n, while $\mu_{eFOV}$ and $\mu_{gt}$ are the mean values of the extended image and the ground truth image. The variances of the extended image and the ground truth image are represented by ${\mu \;}_{eFOV}^{2}$ and ${\mu \;}_{gt}^{2}$, respectively, and $\sigma_{eFOV,gt}$ denotes the covariance between the extended image and the ground truth image. Additionally, $c_{1}$ and $c_{2}$ are constants.

## 4. Results

To evaluate the performance of the proposed Prior-FOVNet, we compared it with both classical algorithms and recently developed deep learning models. The first algorithm is symmetrical data padding in the sinogram domain, known for its high efficiency and straightforward implementation. The second method is based on the WCE model for sinogram extrapolation, as described in Section 2.1. The third model is the plug-and-play (PnP) method that uses the FBPConvNet for truncation correction. Additionally, the eFOV reconstruction using a Unet model was included for comparison.

### 4.1. Results of Synthetic MVCT Images

**Figure 7:**
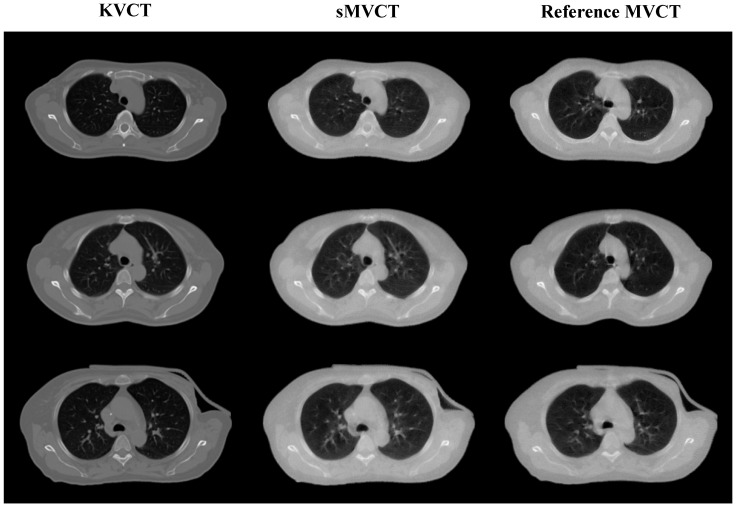
The synthetic MVCT images converted from KVCT. The first column displays the KVCT images of the same patient, while the third column presents the untruncated reference MVCT images. The second column shows the synthetic MVCT images generated through the TransNet.

Figure 7 shows the results of synthesized MVCT images derived from KVCT images, along with the corresponding real MVCT images. The first column displays the planning KVCT images with a large field of view, the second column shows the synthesized MVCT images, and the third column presents the MVCT images, which serve as references. The results demonstrate that the synthesized MVCT images, generated from KVCT using the TransNet method, effectively preserve the anatomical structures of the KVCT while accurately capturing the essential features of the MVCT.

**Figure 8:**
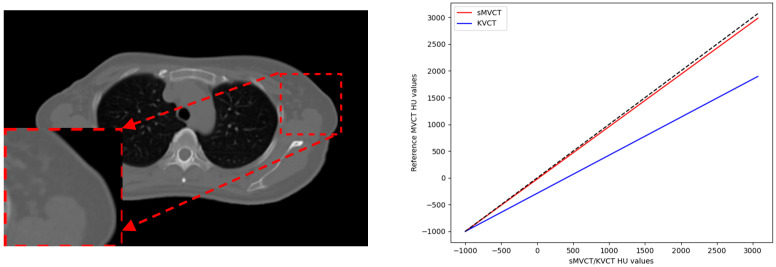
The first column presents the KVCT images included in the linear regression analysis, along with their magnified ROI regions. The second column displays the results of the linear regression analysis, comparing the HU value accuracy within the ROI between the KVCT images, the synthesized MVCT, and the ground truth MVCT. The gray dashed line represents the ideal linear relationship with an intercept of 0 and a slope of 1.

Figure 8 presents the linear regression analysis in terms of the HU value accuracy between the synthesized MVCT image and the real MVCT within an ROI. The results indicate a high degree of similarity between these two datasets. The slope of the regression line between sMVCT and the reference MVCT (red line) is 0.98, with an intercept of −21.26. In contrast, the slope between KVCT and the reference MVCT (blue line) is 0.70, with an intercept of −288.56.

### 4.2. Results of Simulated Data

**Figure 9:**
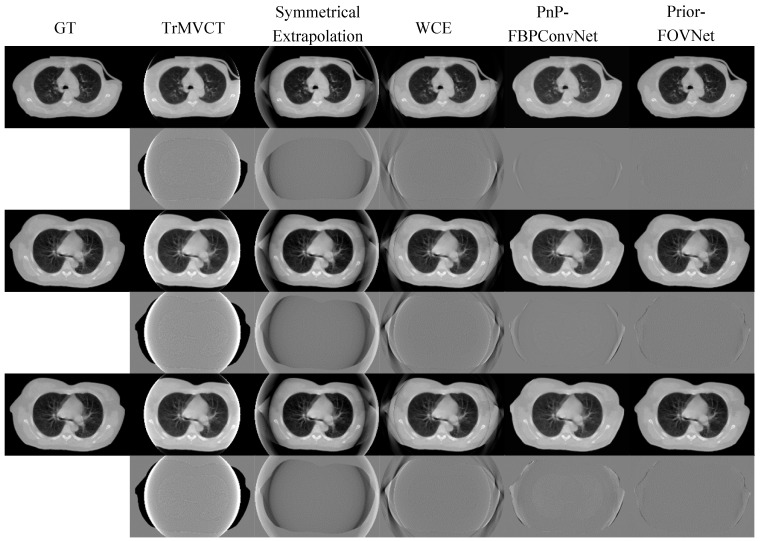
Comparison of thoracic MVCT images corrected by different methods: The first column shows the ground truth, followed by truncated MVCT images with a limited FOV in the second. The third to sixth columns display results from symmetrical extrapolation, water cylinder extrapolation, PnP-FBPConvNet, and Prior-FOVNet, respectively. The display window for each different image is adjusted individually to optimize the interpretation of the visual results.

**Figure 10:**
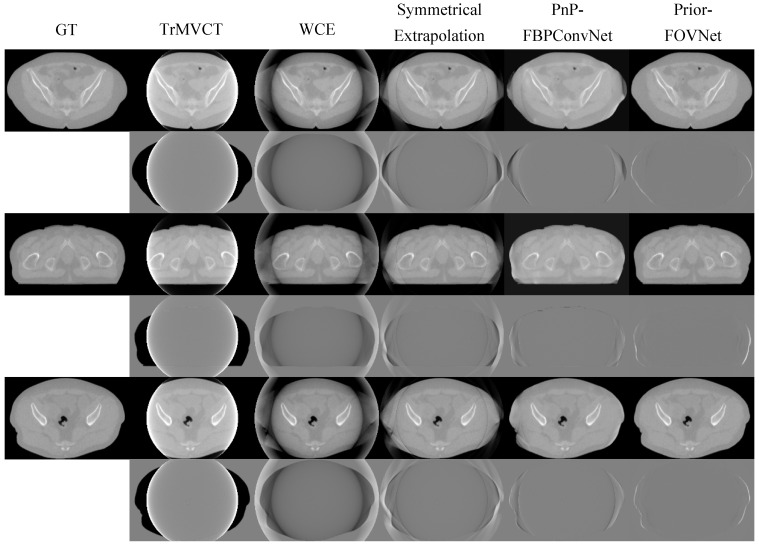
Comparison of pelvic MVCT images corrected by different methods: The first column shows the ground truth, followed by truncated MVCT images with a limited FOV in the second. The third to sixth columns display results from symmetrical extrapolation, water cylinder extrapolation, PnP-FBPConvNet, and Prior-FOVNet, respectively. The display window for each different image is adjusted individually to optimize the interpretation of the visual results.

The comparison of corrected MVCT images generated by different models and their respective difference maps with the ground truth image is shown in Figure 9 (thorax) and Figure 10 (pelvis). The results highlight the performance of each model in terms of artifact correction and image reconstruction. The difference maps provide a visual representation of the residual errors between the predicted and ground truth images, allowing for a qualitative assessment of each model’s ability to restore anatomical details and reduce artifacts. The truncated images exhibit blooming artifacts and missing structural information. Errors are evident in the extrapolated regions produced by both the Symmetric Extrapolation and WCE methods, with anatomical details lost and reconstruction errors and noise outside the FOV. In the difference map for the Symmetric Extrapolation method, numerous bright areas appear along the edges, accompanied by noticeable intensity discrepancies. In comparison, WCE performed better overall, especially in preserving tissue contours. The images produced by PnP-FBPConvNet, though successful in removing erroneous tissues outside the extended FOV, did not fully restore continuity within the eFOV, leading to some deviations from the ground truth. The difference map revealed that the body contours did not align well with the ground truth. In contrast, the Prior-FOVNet method, which incorporates prior knowledge about material and object support, produces images with fewer artifacts and smaller deviations from the ground truth, as evidenced by the minimal differences observed in its difference maps.

**Figure 11:**
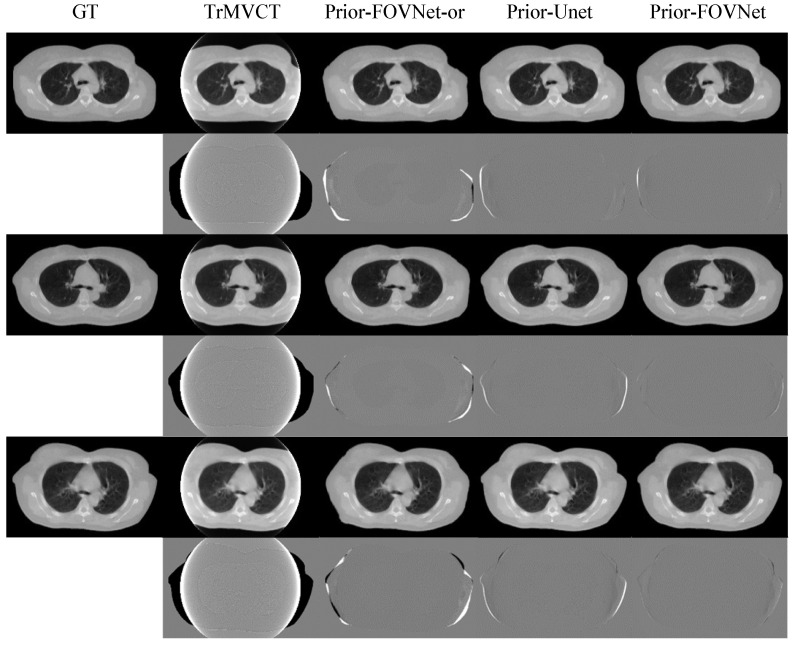
Comparison of the corrected MVCT images generated by the models and their corresponding difference maps with the ground truth image. The display window for each different image is adjusted individually to optimize the interpretation of the visual results.

To explore the necessity of the initial correction for truncation artifacts, as presented in Section 2.1, we tested inputting the original uncorrected MVCT image along with the sMVCT image into the FOV-Net. The corresponding results are shown in the column labeled “Prior-FOVNet-or”. Additionally, to investigate the effectiveness of the use of the Swin Transformer module, we input the pre-corrected image and sMVCT image into a U-Net network, with the results displayed in the column labeled “Prior-Unet”. Figure 11 compares the MVCT image generation results from the Prior-FOVNet and Prior-Unet models. The second, fourth, and sixth rows display the difference maps, which were calculated by subtracting the predicted images from the ground truth MVCT images. In the extended-FOV region of the images predicted by the Prior-FOVNet-or model, noticeable deformations in anatomical structures can be observed. The corresponding difference maps highlight bright and dark areas, indicating significant deviations in body contours and anatomical details different from the ground truth. While the Prior-Unet model produced better image quality with more accurate anatomical structures, some structural distortions persisted. In contrast, the Prior-FOVNet model delivered the best overall image quality, with the smallest deviation from the ground truth.

Table 1 compares the quantitative results of different models on the test dataset. The data extrapolation algorithms, specifically the symmetrical extrapolation and WCE methods, showed significantly higher error rates and lower SSIM scores, underscoring their limitations in reducing artifacts and restoring anatomical details. In contrast, deep learning-based models such as PnP-FBPConvNet demonstrated marked improvements, achieving an MAE of 38.0 ± 3.6 HU and an SSIM of 95.8 ± 1.0%. Our proposed model, Prior-FOVNet, achieved the best overall performance across all metrics. Specifically, Prior-FOVNet obtained the lowest MAE of 23.8 ± 5.6 HU, the lowest RMSE of 50.8 ± 18.4 HU, and the highest SSIM of 97.8 ± 0.6%. These results highlight the superiority of Prior-FOVNet in mitigating truncation artifacts and reconstructing missing structural information.

**Figure 12:**
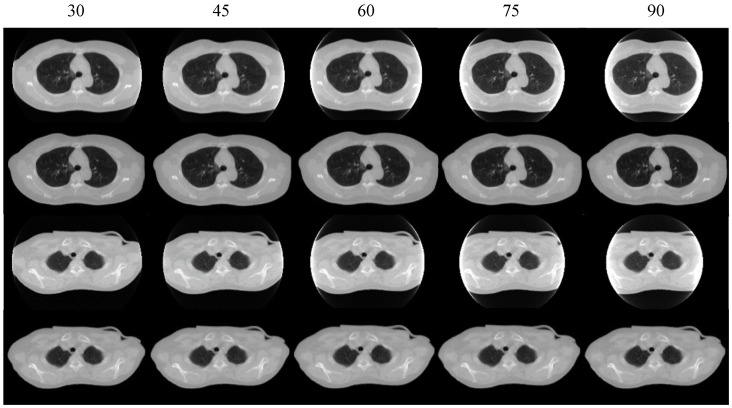
Generalization test results of the model across different truncation ranges. The first to fifth columns represent the results for truncation values of 30, 45, 60, 75, and 90 detector units, respectively.

To assess the robustness of the Prior-FOVNet model, we conducted experiments using varying degrees of truncation. The projection data with a truncation of 30, 45, 60, 75, 90 detector units were employed for experiments. The results are displayed in Figure 12. It can be seen that the model performs excellently on datasets with smaller truncation ranges while still maintaining reasonable performance even with larger truncation ranges. A quantitative evaluation of the model’s performance is provided in Table 2. The results indicate that the model can perform effectively even with larger truncation ranges. Overall, the model demonstrates strong and consistent performance across different truncation levels. The ability of Prior-FOVNet to adapt to a wide range of truncation levels highlights its robust generalization capability, ensuring reliable and effective performance in practical scenarios with varying truncation artifacts.

### 4.3. Results of Clinical Patient Data

**Figure 13:**
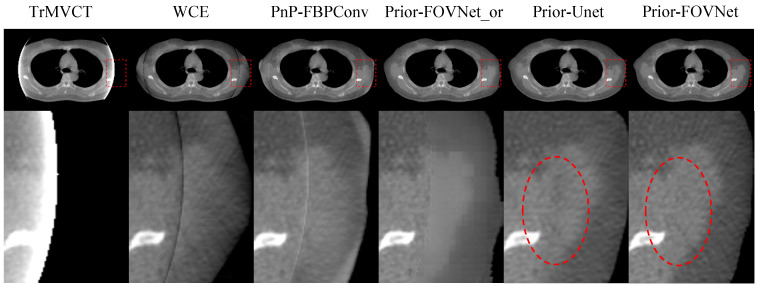
Results of clinical patient data. The bottom row shows the zoomed ROIs of the boxed area in the first row.

**Figure 14:**
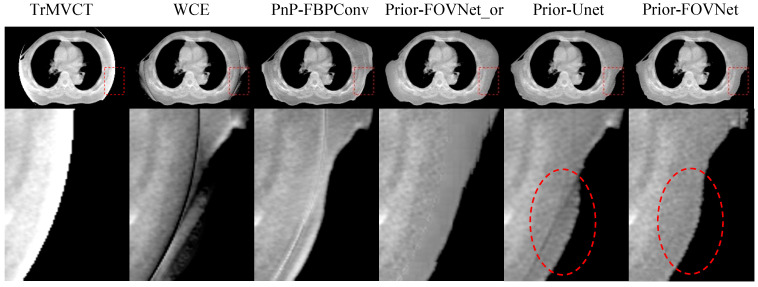
Results of clinical patient data. The bottom row shows the zoomed ROIs of the boxed area in the first row.

Figure 13 and Figure 14 show the results of the models applied to clinical patient datasets. In the first column of Figure 13 and Figure 14, the original truncated MVCT images are displayed with zoomed ROIs, revealing severe blooming artifacts inside the FOV and a complete loss of anatomical structures outside the FOV. The second column depicts the results from WCE, where the anatomical structures and contours outside the FOV are significantly distorted with line artifacts, indicating that the generated content in this region is inaccurate. The third and fourth columns show the outcomes of PnP-FBPConvNet and Prior-FOVNet-or, respectively, where artifacts within the FOV are significantly reduced, and the regions outside the FOV are appropriately reconstructed. However, a distinct seam is observed between the original image within the sFOV and the newly generated areas outside. Upon further inspection, the PnP-FBPConvNet exhibits distortions in anatomical structures outside the FOV, while the Prior-FOVNet-or reveals pronounced texture inconsistencies in the newly synthesized regions. For the generated results of Prior-Unet and Prior-FOVNet, both methods effectively reduce the truncation artifacts and reconstruct anatomical structures outside the sFOV. However, when closely inspecting the regions highlighted by the red circles, it is evident that the results from Prior-Unet exhibit discontinuities at the truncated FOV boundary, while the results from Prior-FOVNet show noticeably smoother transitions.

### 4.4. Ablation Study

**Figure 15:**
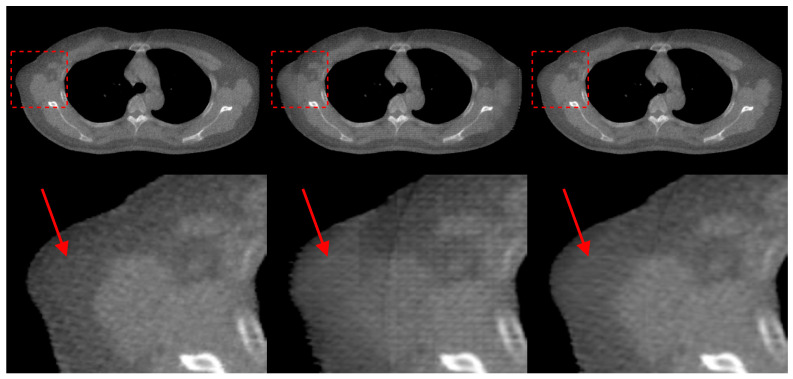
Results of the ablation study on the use of prior images. The first column shows the ground truth MVCT images, the second column presents the correction results from the FOVNet model without a prior image, and the third column displays the correction results from the Prior-FOVNet model with the TransNet module.

In the Prior-FOVNet method, the TransNet is designed to extract material and shape priors from similar KVCT images by converting the KVCT into sMVCT, which serves as prior information to enhance the reliability and stability of the final results. To verify the effectiveness of the reference KVCT images, we removed the image transformation network and input the extrapolated truncated MVCT directly into FOV-Net for truncation correction and FOV extension. We compared the truncation correction results with and without using the reference images, as shown in Figure 15. It can be observed that without the reference images as priors, artificial structures are generated, as indicated by the red arrows. We also conducted a quantitative evaluation of the model’s results, as summarized in Table 3. The model with prior information from the reference images achieved superior performance across all metrics, highlighting the critical role that reference images play in ensuring the reliability and stability of the generated image outside the sFOV.

## 5. Discussion and Conclusions

In this study, we proposed Prior-FOVNet, specially designed to address truncation artifacts and extend the field of view in MVCT images by leveraging prior information from KVCT images. The experimental results demonstrated that our method outperformed traditional extrapolation and recently developed deep learning-based models by producing more accurate eFOV reconstructions with fewer artifacts. Specifically, Prior-FOVNet achieved significantly lower mean absolute error (MAE) and root mean square error (RMSE) while maintaining a higher structural similarity index (SSIM) compared to other models, highlighting its capability to reconstruct details outside the original sFOV.

In Prior-FOVNet, the correction process begins with sinogram domain extrapolation with the WCE model to reduce the blooming effect near the edges of the limited field of view. This initial correction provides a cleaner baseline for the FOV-Net, ensuring that subsequent stages can focus on refining anatomical details without being affected by these distortions. Without this initial step, abrupt intensity changes at the boundaries of the sFOV may lead to artificial structures and intensity changes. Moreover, the initial correction with the WCE model provides an initial object shape support to the target MVCT images. The necessity of this step is evident from the results shown in Figure 13 and Figure 14, where omitting the initial correction led to the generation of inconsistent structures, as highlighted by the zoomed ROIs. When the initial correction is applied, the model effectively extends the field of view and corrects truncation artifacts without introducing new distortions.

The inclusion of prior images in Prior-FOVNet is crucial for addressing the challenges posed by severe projection data truncation, which traditional extrapolation techniques often fail to address comprehensively. KVCT images of the same patient provide complete structural information and serve as a reliable reference, which is crucial when correcting the truncated MVCT images where anatomical structures are missing or distorted due to the limited sFOV. To further enhance this process, the conversion of KVCT into synthesized MVCT is necessary for aligning the intensity distributions between the two modalities. By employing a contrastive learning-based GAN (TransNet) to transform KVCT images into sMVCT, the model ensures that the synthesized images maintain similar intensity characteristics to real MVCT images while preserving anatomical details from KVCT. This allows the model to directly use sMVCT as a prior for correcting truncated regions in MVCT, effectively overcoming the challenges associated with modality differences. Additionally, the model’s reliance on prior knowledge from KVCT images plays a crucial role in its generalization. This use of external priors helps mitigate the common issue of overfitting, which is often a concern in deep learning models trained on limited datasets.

In this work, a Swin Transformer-based U-Net was employed for field-of-view extension. The Swin Transformer’s window-based mechanism effectively captures both local and global features through Window-based Multi-Head Self-Attention (W-MSA) and Shifted Window-based Multi-Head Self-Attention (SW-MSA). This enables the network to process fine-grained details while preserving the broader structural context of the image, allowing it to maintain structural coherence even in the presence of minor structural discrepancies between the target MVCT and sMVCT. Furthermore, the skip connections in the U-Net structure help preserve low-level features throughout the encoding and decoding processes.

The generalization of the proposed Prior-FOVNet was rigorously evaluated across multiple truncation levels and patient datasets, and the results demonstrate that the model is adaptable to different scenarios. Firstly, the model’s ability to handle varying degrees of truncation was tested using projection data with detector truncations ranging from 30 to 90 units. The results showed that Prior-FOVNet maintained strong performance across all truncation levels, consistently producing high-quality reconstructions with minimal errors. As highlighted in Table 2, even at higher truncation levels, where anatomical details are more challenging to recover, the model achieved relatively low MAEs and RMSEs, while maintaining a high SSIM. This suggests that the model is robust in handling severe data loss, effectively restoring the missing structures outside the field of view even under challenging conditions. Furthermore, the model was tested on clinical patient datasets, as shown in Figure 13 and Figure 14, which indicate that Prior-FOVNet successfully corrected truncation artifacts and extended the FOV across diverse clinical cases. Importantly, the model produced smooth transitions between the original and extended-FOV regions, avoiding the texture discontinuities and structural distortions that are often seen in alternative methods like water cylinder extrapolation or plug-and-play models.

Despite the promising results demonstrated by Prior-FOVNet in correcting MVCT truncation artifacts and extending the field of view, several limitations should be acknowledged. Firstly, the model’s reliance on KVCT images as priors makes it inherently dependent on the availability and quality of these images. Poor image quality in the KVCT may affect the accuracy of the synthesized MVCT and subsequently hinder the correction process. Additionally, residual errors can still be found in some cases, particularly in the boundary regions. The contributing factor should be the incomplete registration between the prior and target images. Another limitation lies in the computational complexity of the proposed approach. Incorporating a Swin Transformer-based architecture for the FOV-Net increases the number of parameters significantly, which may lead to longer training times and higher computational resource requirements compared to the conventional models. Finally, this study primarily focuses on truncated MVCT images from specific imaging geometry, and the model’s performance in other cases such as off-center scans, where the nature of truncation may differ, remains to be thoroughly investigated.

## Figures and Tables

**Table 1:** Comparison of the mean absolute error (MAE in HU), root mean square error (RMSE in HU), and structural similarity index (SSIM in %) for the MVCT images corrected by each model across all test data. Values are presented as mean ± standard deviation, based on a total of 255 test cases.

	Symmetrical Extrapolation	WCE	PnP-FBPConv	Prior-FOVNet-or	Prior-Unet	Prior-FOVNet
MAE	189.3 ± 52.5	93.5 ± 33.5	38.0 ± 3.6	60.8 ± 28.4	40.3 ± 10.5	23.8 ± 5.6
RMSE	277.1 ± 72.9	209.2 ± 64.3	61.6 ± 11.3	182.4 ± 76.2	100.6 ± 42.6	50.8 ± 18.4
SSIM	85.6 ± 3.4	89.0 ± 4.2	95.8 ± 1.0	94.6 ± 1.6	95.4 ± 1.1	97.8 ± 0.6

**Table 2:** Quantitative evaluation of the model’s generalization performance across different truncation ranges.

	30	45	60	75	90
MAE (HU)	24.16	22.27	19.96	35.20	70.30
RMSE (HU)	56.18	34.88	31.65	95.23	131.98
SSIM (%)	98.13	98.15	98.00	96.47	94.75

**Table 3:** Quantitative evaluation results of ablation study.

	MAE (HU)	RMSE (HU)	SSIM (%)
FOVNet (without TransNet)	45.95	107.66	94.90
Prior-FOVNet (with TransNet)	26.49	77.00	97.18

## Data Availability

Data are contained within the article.
